# Exposing the gaps in awareness, knowledge and estimation of risk for anal cancer in men who have sex with men living with HIV: a cross-sectional survey in Australia

**DOI:** 10.7448/IAS.18.1.19895

**Published:** 2015-03-30

**Authors:** Jason J Ong, Marcus Chen, Andrew Grulich, Sandra Walker, Meredith Temple-Smith, Catriona Bradshaw, Suzanne M Garland, Richard Hillman, David Templeton, Jane Hocking, Beng Eu, BK Tee, Christopher K Fairley

**Affiliations:** 1 Melbourne School of Population and Global Health, University of Melbourne, Melbourne, Australia; 2 Melbourne Sexual Health Centre, Alfred Health, Melbourne, Australia; 3 Central Clinical School, Monash University, Melbourne, Australia; 4 Kirby Institute, University of New South Wales, Kensington, Australia; 5 General Practice and Primary Health Care Academic Centre, University of Melbourne, Melbourne, Australia; 6 Department of Obstetrics and Gynaecology, University of Melbourne, Melbourne, Australia; 7 Westmead Clinical School, University of Sydney, Sydney, Australia; 8 Prahran Market Clinic, Melbourne, Australia; 9 The Centre Clinic, Victorian AIDS Council, Melbourne, Australia

**Keywords:** HIV, men who have sex with men, anal cancer, knowledge, risk

## Abstract

**Introduction:**

The incidence of anal cancer is significantly higher in men who have sex with men (MSM) living with HIV when compared to the general population. We aimed to assess their awareness, knowledge and perceived level of personal risk for anal cancer to help inform educational strategies targeting this group.

**Methods:**

A cross-sectional study of 327 HIV positive MSM in Melbourne, Australia, attending clinical settings (a sexual health centre, tertiary hospital HIV outpatients and high HIV caseload general practices) completed a written questionnaire in 2013/14. Poor knowledge was defined as those who had never heard of anal cancer, or scored 5 or less out of 10 in knowledge questions amongst those who reported ever hearing about anal cancer. Underestimation of risk was defined as considering themselves as having the same or lower risk for anal cancer compared to the general population.

**Results:**

Of 72% (95% confidence interval (CI): 67–77) who had heard of anal cancer, 47% (95% CI: 41–53) could not identify any risk factors for anal cancer. Of total men surveyed, 51% (95% CI: 46–57) underestimated their risk for anal cancer. Multivariate analysis showed that men who underestimated their risk were older (OR 1.04 (per year increase in age), 95% CI: 1.01–1.07), had poor anal cancer knowledge (OR 2.06, 95% CI: 1.21–3.51), and more likely to have ever had an anal examination (OR 2.41, 95% CI: 1.18–4.93). They were less likely to consult a physician if they had an anal abnormality (OR 0.54, 95% CI: 0.31–0.96), to have had receptive anal sex (OR 0.12, 95% CI: 0.02–0.59) or speak English at home (OR 0.28, 95% CI: 0.09–0.90).

**Conclusions:**

This survey of MSM living with HIV demonstrated limited awareness, knowledge level and estimation of risk for anal cancer. Further educational and public health initiatives are urgently needed to improve knowledge and understanding of anal cancer risk in MSM living with HIV.

## Introduction

The causes of mortality for people living with HIV in developed countries are changing, with fewer people dying from AIDS, and more from non-AIDS-related comorbidities [1]. Consistent with this change, there has recently been a shift in focus from treating acute illnesses towards preventive health and management of multiple morbidities associated with an ageing HIV population. With access to highly active antiretroviral therapy, the expectation is that well-managed patients will live long and healthy lives [2].

Whilst there has been much research on managing cardiovascular, renal, bone and neurological complications for people living with HIV, there is relatively less research focused on prevention of non-AIDS defining malignancies. This is an important area of research as non-AIDS defining malignancies are causing an increased proportion of mortalities over time [1].

In Australia, anal cancer is now the most common non-AIDS defining malignancy in people living with HIV [3]. In particular, the group at highest risk for anal cancer is men who have sex with men (MSM) living with HIV. They have multiple risk factors including high prevalence and persistence of high-risk anal human papillomaviruses (HPVs) [4,5], and high rates of smoking [6] in the context of a substantially increased life expectancy and chronic immune dysregulation [2]. It is estimated that anal cancer incidence rates among MSM living with HIV range from 46 to 131 per 100,000 person years [4,7]. However, in most settings, few MSM living with HIV are currently being screened for anal cancer or its precursors [8,9].

To improve anal cancer screening behaviours, it is important to determine a patient's awareness and understanding of the condition for which they are being screened. Health literacy, an individual's ability to read, understand and use information necessary to obtain adequate healthcare, has been shown to affect cancer screening behaviours [10] and low health literacy may be associated with poorer health [11]. Surveys of anal cancer awareness and knowledge level have been conducted for MSM without HIV [12,13] but, to our knowledge, this study is the first to present findings from a large group of MSM living with HIV. Given that the highest risk for anal cancer is MSM living with HIV, it is important to determine the current awareness, knowledge and estimation of risk for anal cancer, to inform educational strategies targeting this group.

## Methods

As part of a larger trial (the ACE study) [14], MSM living with HIV completed a questionnaire containing demographic details (e.g. age, smoking status), HIV status (current CD4 and viral load, CD4 nadir), and sexual practices (lifetime and within 6 months). They were also asked a series of questions to evaluate awareness, knowledge of anal cancer and HPV, derived from a questionnaire administered to MSM [12]. A correct answer for a risk factor for anal cancer included smoking tobacco, and/or any sexual behaviours that may increase the risk of acquiring anal HPV (e.g. receptive anal sex and/or high numbers of sexual partners [15]). To evaluate personal risk for anal cancer, we asked men to estimate their risk for developing anal cancer compared to a heterosexual male without HIV of the same age (“much higher than average,” “higher than average,” “about the same,” “lower than average,” “much lower than average”).

A more detailed account of the recruitment strategy is described elsewhere [16]. Briefly, men were recruited from three types of clinical settings – a sexual health centre, a tertiary hospital HIV outpatient clinic and two high case load general practices. All services see a large number of clients with HIV and are the main services accessed by individuals with HIV in Victoria with the exception of one other large general practice that was not included in this study. Men were eligible for inclusion if they had adequate English and comprehension skills to give informed consent, were MSM living with HIV, over 35 years old and had no past diagnosis of anal cancer. The starting age of 35 years was chosen because it is rare to have anal cancer detected below this age [17]. There was no upper age limit.

### Statistical analysis

Patient demographics were analyzed descriptively. The five-point response for risk estimation was collapsed into two categories, e.g. “much higher than average, higher than average” and “about the same/lower than average/much lower than average.” Ninety five percent confidence intervals (CIs) were calculated for proportions using the modified Wald method. To test statistical significance between groups, chi-square and Wilcoxon–Mann–Whitney tests were used for categorical data. We conducted multivariate logistic regression analyses to identify factors associated with men who had not heard of anal cancer, had poor knowledge of anal cancer and had underestimated their personal risk for anal cancer. Variables were chosen for inclusion in the models on the basis of the likelihood ratio test. All analyses were performed using the statistical software, STATA (StataCorp. 2013. *Stata Statistical Software: Release 13*. College Station, TX: STatCorp LP.).

This research was approved by the Alfred Health Human Ethics Committee (Project 246/12).

## Results

### Demographics

A total of 327 MSM living with HIV were recruited from December 2012 to November 2013. The average recruitment rates of potentially eligible men by site were 45% for the sexual health centre, 11% for general practice and 5% for hospital (*p* value for difference <0.001). The demographics are summarized in Table 1. There were no statistically significant differences for all demographic variables between men from each site. Most men (89%, 95% CI: 85–92) were comfortable discussing anal cancer, and 85% (95% CI: 81–89) thought it was an important topic to discuss with their HIV physician.

**Table 1:** Demographics of participants

	*n* (%)
Born in Australia	215 (69)
Speaks English at home	294 (94)
Education	
Primary	5 (2)
Secondary	101 (32)
Technical and further education	92 (29)
University	70 (22)
Postgraduate	43 (14)
Employment	
Work full time	152 (49)
Work part time	49 (16)
Unemployed	29 (9)
Retired	52 (17)
Other (student, home duties, unspecified)	26 (8)
Annual household income ($AUS)	
1–59,999	121 (42)
60,000–99,999	64 (22)
>100,000	58 (20)
no answer	47 (16)
Healthcare card holder^a^	108 (35)
Private health insurance	119 (39)
Smoker	
Current	100 (32)
Ex-smoker	107 (34)
Never smoked	104 (33)
Anal symptoms in last 3 months	145 (48)
Viral load^b^ <50 copies/mL	252 (77)
Currently on antiretrovirals	288 (95)
Mean age (years (sd))	51 (9)
Mean years living with HIV (years (sd))	13 (8)
Mean CD4 (cells per mm^3^, (sd))	630 (265)

a Healthcare cards are given to Australian residents with a lower income to be able to access cheaper medicines and medical costs;

b Based on latest blood test at recruitment.

### Knowledge of anal cancer

Over a quarter (28%, 95% CI: 23–33) of men had never heard of anal cancer. Physicians were the overwhelmingly main source of information relied upon by participants (Table 2), yet only 34% (95% CI: 28–39) of patients reported that a health professional had ever discussed anal cancer with them. Only a minority of men would rely on the internet, magazines, television, community organizations and brochures as their source of information about anal cancer.

**Table 2:** What sources have you relied on for information about anal cancer? (*n*=309)

Source	*n*	Percentage (95% CI)
Physician	198	64 (59–69)
Internet	63	20 (16–25)
Family and friends	61	20 (16–25)
Magazines	49	16 (12–20)
Television	46	15 (11–19)
Other (community organization, brochures)	25	8 (6–12)
Practice nurse	18	6 (4–9)

Table 3 summarizes factors associated with those who had never heard of anal cancer compared to those who had. The only independent correlates of never having heard of anal cancer were not having a health professional discuss anal cancer with them and not feeling it was important to discuss anal cancer. Our final model was adjusted *a priori* for speaking English at home and education level, given their known association with health literacy [11].

**Table 3:** Univariate and multivariate analysis of factors associated with not having heard of anal cancer

Factor	Never heard of anal cancer *n* (%)	Heard of anal cancer *n* (%)	OR (95% CI)	aOR (95% CI)
Age (per year increase)	–	–	0.97 (0.94–1.00)	–
Receiving 3-monthly HIV care				
No	12 (18)	68 (29)	1	
Yes	55 (82)	168 (71)	1.86 (0.94–3.68)	–
Change in bowel habits in last 3 months				
No	48 (72)	194 (82)	1	
Yes	19 (28)	42 (18)	1.83 (0.98–3.42)	–
Ever had an anal examination				
No	18 (27)	33 (14)	1	
Yes	49 (73)	203 (86)	0.44 (0.23–0.85)	–
Health professional discussed about anal cancer				
No	61 (91)	134 (58)	1	1
Yes	6 (9)	97 (42)	0.14 (0.06–0.33)	0.14 (0.06–0.33)
Important to discuss about anal cancer				
No	45 (67)	123 (52)	1	1
Yes	22 (33)	113 (48)	0.53 (0.30–0.94)	0.45 (0.24–0.82)
Education level – university or above				
No	48 (72)	145 (61)	1	1
Yes	19 (28)	91 (39)	0.63 (0.35–1.14)	0.58 (0.30–1.11)
Speak English at home				
No	6 (9)	16 (7)	1	1
Yes	61 (91)	220 (93)	0.74 (0.28–1.97)	0.43 (0.14–1.31)

aOR=adjusted odds ratio.

Table 4 summarizes knowledge about anal cancer and HPV in those who stated they have heard of anal cancer. Of these men, 47% (95% CI: 41–53) could not identify any risk factors for anal cancer and 46% (95% CI: 39–52) scored 5 or less out of 10 in the knowledge questions. There were no statistically significant differences in knowledge between men recruited from the sexual health centre, tertiary hospital HIV outpatient and general practices.

**Table 4:** Ten knowledge questions for those who have heard of anal cancer (*N*=236)

Question	*n*	Percentage correct (95% CI)
Anal cancer rates are rising for MSM	88	37 (31–44)
Identified at least one risk factor for anal cancer	125	53 (47–59)
Only MSM living with HIV are at risk for anal cancer (false)	166	70 (64–76)
Heard of HPV	180	76 (70–81)
HPV can cause anal cancer	133	56 (50–63)
HPV is a sexually transmitted infection (STI)	112	47 (41–54)
HPV is the most common STI	44	19 (14–24)
HPV affects both men and women	132	56 (50–62)
Condoms do not always prevent HPV transmission	59	25 (20–31)
HPV can cause warts	118	50 (44–56)

To evaluate if there were factors associated with differences in knowledge, we defined poor knowledge as men who had never heard of anal cancer before or scored 5 or less in the 10 knowledge questions asked. Table 5 summarizes the characteristics of those with poor knowledge, who were older and less likely to ever self-examine their anus, to have had a health professional discuss anal cancer with them, to complete tertiary education and to speak English at home.

**Table 5:** Univariate and multivariate analysis of characteristics associated with men with poor knowledge about anal cancer (i.e. ≤5 out of 10 in knowledge questions)

Factor	Poor knowledge *n* (%)	Good knowledge *n* (%)	OR (95% CI)	aOR (95% CI)
Age (per year increase)	–	–	1.03 (1.00–1.06)	1.03 (1.00–1.07)
Satisfied with HIV doctor				
No	34 (16)	10 (9)	1	–
Yes	180 (84)	103 (91)	0.51 (0.24–1.08)	
Ever received an anal swab				
No	35 (18)	11 (12)	1	
Yes	162 (82)	101 (89)	0.55 (0.27–1.11)	–
Ever self-examined				
No	148 (75)	57 (50)	1	1
Yes	48 (25)	56 (50)	0.33 (0.20–0.54)	0.32 (0.19–0.55)
Health professional discussed about anal cancer				
No	146 (76)	57 (50)	1	1
Yes	47 (24)	56 (50)	0.33 (0.20–0.54)	0.32 (0.19–0.55)
Ever had receptive anal sex				
No	15 (8)	2 (2)	1	
Yes	176 (92)	109 (98)	0.22 (0.05–0.96)	–
Completed tertiary education				
No	152 (71)	62 (55)	1	1
Yes	62 (29)	51 (45)	0.50 (0.31–0.80)	0.50 (0.20–0.85)
Speak English at home				
No	32 (15)	5 (4)	1	1
Yes	182 (85)	108 (96)	0.26 (0.10–0.70)	0.28 (0.09–0.90)

### Underestimation of anal cancer risk

Over half of the participants (51%, 95% CI: 46–57) stated their risk for anal cancer as “about the same,” “lower” or “much lower” compared to an average man their age without HIV. There was no difference in personal risk factors for anal cancer between men who underestimated their risk, compared with those who did not. Similar proportions were current smokers (*p*=0.289) and both groups had a similar mean number of lifetime receptive anal sexual partners (*p*=0.929). There was a slight difference in age, with men of an older mean age being more likely to underestimate their anal cancer risk (52 vs. 50 years old, *p*=0.017).

In a multivariate logistic regression model, factors associated with men who underestimated their risk (Table 6) were: having poor anal cancer knowledge and ever having had an anal examination by a physician. Men who underestimated their risk were less likely to: consult a physician if they had an anal abnormality within the last 3 months, had ever had receptive anal sex and speak English at home. There were no differences in number of years living with HIV (*p*=0.258), being engaged in 3-monthly HIV care (*p*=0.608), having had a discussion with a health professional about anal cancer (*p*=0.974), comfort discussing anal cancer (*p*=0.119), or thinking anal cancer was an important topic to discuss with their HIV physician (*p*=0.930).

**Table 6:** Univariate and multivariate analysis of characteristics associated with men who underestimated their risk of anal cancer

Factor	Underestimated risk *n* (%)	Correctly estimated risk *n* (%)	OR (95% CI)	aOR (95% CI)
Age (per year increase)	–	–	1.04 (1.01–1.07)	1.04 (1.01–1.07)
Born in Australia				
No	57 (38)	34 (24)	1	
Yes	95 (62)	110 (76)	0.52 (0.31–0.85)	–
Poor knowledge				
No	43 (28)	69 (48)	1	1
Yes	109 (72)	75 (52)	2.33 (1.44–3.77)	2.06 (1.21–3.51)
Had anal abnormality in last 3 months				
Did not consult physician	113 (75)	91 (64)	1	1
Consulted a physician	38 (25)	51 (36)	0.60 (0.36–0.99)	0.54 (0.31–0.96)
Ever had an anal swab				
No	28 (18)	13 (9)	1	
Yes	124 (82)	130 (91)	0.44 (0.22–0.89)	–
Ever had an anal examination				
No	19 (12)	30 (21)	1	1
Yes	133 (88)	113 (79)	1.86 (0.99–3.48)	2.41 (1.18–4.93)
Ever had partner examine their anus				
No	144 (95)	127 (89)	1	
Yes	8 (5)	16 (11)	0.44 (0.18–1.06)	–
Ever had receptive anal sex				
No	15 (10)	2 (1)	1	1
Yes	137 (90)	140 (99)	0.13 (0.03–0.58)	0.12 (0.02–0.59)
Completed tertiary education				
No	100 (66)	87 (60)	1	1
Yes	52 (34)	57 (40)	0.79 (0.49–1.27)	0.76 (0.44–1.30)
Speak English at home				
No	18 (12)	4 (3)	1	1
Yes	134 (88)	140 (97)	0.21 (0.07–0.64)	0.18 (0.05–0.62)

## Discussion

In our study we found that there were gaps in awareness (28% had not heard of anal cancer) and even among those who had heard of anal cancer, nearly half of men had poor knowledge. We also found that half of men underestimated their personal risk for anal cancer. Given that MSM living with HIV are one suggested target group for anal cancer screening [18] and anal cancers are currently diagnosed late [19], strategies to improve screening or encourage early clinical presentation will need to improve knowledge and understanding of risk for anal cancer in this group.

If we improve understanding of anal cancer, there might be a number of health benefits. Firstly, greater awareness of anal cancer symptoms may lead to earlier presentation to a physician compared to the current mean duration of 22 weeks before men with anal cancer sought medical attention in one recent study [19]. If MSM living with HIV can be taught to present earlier to a health professional if they have symptoms or signs suggestive of anal cancer, there is greater likelihood of earlier diagnosis and therefore reduced associated morbidity and mortality [20]. Secondly, MSM living with HIV who understands their risk for anal cancer may be more accepting of screening if it becomes recommended. We found that men who had never had anal receptive sex or had an anal examination by a physician underestimated their *on-going* risk, stating they had the same or lower risk for anal cancer than a heterosexual male without HIV. Studies have shown a high prevalence of anal HPV in MSM living with HIV [4], even in those who reported never having receptive anal sex [21]. Given the absence of any studies demonstrating that anal cancer screening using digital ano-rectal examination is effective in reducing anal cancer related morbidity and mortality [22], having a normal anal examination may not equate to lack of on-going risk for anal cancer. Patients should understand their on-going risk for anal cancer and remain vigilant for potential symptoms of anal cancer such as bleeding, discharge, pain and lumps. Correct estimation of cancer risk has been shown to improve health-seeking behaviours for cervical cancer [23] and colorectal cancer [24]. Indeed a large study of more than 20,000 people surveyed for likelihood of participation in colorectal screening found that the lack of awareness of risk was a major barrier to screening [25]. Furthermore, poor knowledge has been associated with underestimating the risk for colorectal cancer [26]. Increasing the knowledge levels of risk for anal cancer may potentially lead to greater participation in screening strategies.

There are a number of findings from our study that suggest that HIV physicians may play an important role in educating their patients about anal cancer. Firstly, we found that patients largely relied upon their HIV physician as their source of information about anal cancer. In addition, awareness of anal cancer in the sample population was significantly associated with having had a health professional discuss anal cancer with them. Given the poor knowledge and underestimation of risk, the current level of information provided by health professionals appears to be inadequate.

Previous research has used a number of strategies to increase knowledge transfer between patients and health professionals [27]. Whilst health professionals are an important source of health information [28], there are increasing time-pressures for HIV physicians to manage the multiple comorbidities that may be found in people living with HIV. There is potential to explore alternate modes of health information transfer, such as social media for health promotion [29].

A limitation of this study was that men were recruited from HIV clinics using convenience sampling. We noted a large variation in recruitment rates according to clinical site suggesting our findings may not be representative of all MSM living with HIV who attend an HIV clinic. We were unable to collect any information comparing those who participated with those who did not, so we cannot rule out selection bias. However, our study population showed similarities in key demographics with another large representative cohort of people living with HIV in Australia (i.e. mean age of 49 years, majority (78%) were Australian born, 97% spoke English at home, 30% were current smokers, and 58% were in full employment) [30]. Given that our sample contained men already engaged in HIV care who were interested in participating in an anal cancer screening study, it is possible that their knowledge and perception of risk for anal cancer may be better than MSM living with HIV not engaged in HIV care. Recruitment from clinical settings may have influenced men's estimate of their anal cancer risk as the study consent process contained information about MSM living with HIV having a higher risk for anal cancer. Despite being provided with this information at recruitment, we still found half of men underestimated their own personal risk for anal cancer. Also, by excluding men who were not fluent in English, our sample population selected for those with better health literacy. However, it is unlikely that men who are not involved in a study, not engaged in HIV care or are non-English speakers would have greater awareness of their risk of anal cancer. It was interesting to note that we did not find any statistically significant associations between knowledge levels and variables that may be a surrogate for greater opportunities to hear about anal cancer (i.e. HIV duration, low CD4 counts or referral source (clinic type) as a marker for disease severity). This may reflect the general absence of discussion about anal cancer amongst HIV physicians currently. A second limitation relates to the questionnaire. Although we derived our knowledge questions from another study [12], it may be beneficial for additional questions to be included in a future questionnaire, for example symptom awareness for anal cancer. Rather than relying on one question for risk perception for anal cancer, a future questionnaire might include a more sophisticated assessment for risk perception, such as those used for other cancers [31]. The current questionnaire has been able to provide useful broad level information on a range of variables affecting estimation of risk. However, in common with most quantitative research, it lacks the ability to provide an explanatory model. For this reason, we recommend that further qualitative research to elucidate the relationship between risk and knowledge is necessary.

## Conclusions

There are substantial gaps in awareness, knowledge level and estimation of risk for anal cancer in MSM living with HIV, who are the group at highest risk for anal cancer. Whilst the majority of men relied on a health professional for information about anal cancer, there is currently poor information transfer, with nearly half of men scoring poorly on the knowledge test and underestimating their risk for anal cancer. Given that MSM living with HIV are one suggested target group for anal cancer screening and anal cancers are currently diagnosed late, strategies to improve screening or encourage early clinical presentation will need to improve knowledge and understanding of risk for anal cancer in this group.
